# Genetic Testing for Inherited Cardiac Arrhythmias: Current State-of-the-Art and Future Avenues

**DOI:** 10.19102/icrm.2018.091102

**Published:** 2018-11-15

**Authors:** Robyn J. Hylind, Stephanie F. Chandler, Jonathan R. Skinner, Dominic J. Abrams

**Affiliations:** ^1^Inherited Cardiac Arrhythmia Program, Department of Cardiology, Boston Children’s Hospital and Harvard Medical School, Boston, MA, USA; ^2^Green Lane Paediatric and Congenital Cardiac Services, Starship Children’s Hospital, Auckland, New Zealand; ^3^Department of Paediatrics, Child and Youth Health, The University of Auckland, Auckland, New Zealand

**Keywords:** Catecholaminergic polymorphic ventricular tachycardia, genetics, long QT syndrome, pathogenicity

## Abstract

The seminal discovery that sequence variation in genes encoding cardiac ion channels was behind the inherited cardiac arrhythmic syndromes has led to major advances in understanding the functional biological mechanisms of cardiomyocyte depolarization and repolarization. The cost and speed with which these genes can now be sequenced have allowed for genetic testing to become a major component of clinical care and have led to important ramifications, yet interpretation of specific variants needs to be performed within the context of the clinical findings in the proband and extended family. As technology continues to advance, the promise of therapeutic manipulation of certain genetic pathways grows ever more real.

## Introduction

In the last two decades, genetic testing for inherited cardiac arrhythmias has evolved from gene discovery to an important component of clinical care that is both affordable and returns results in a matter of only a few weeks. The seminal discovery that genetically encoded abnormalities in cardiac ion channels underlie inherited cardiac arrhythmias has allowed for both an improved understanding of the molecular biological processes fundamental to different disease states and also has helped to guide therapeutic strategies. The evolution of patient-specific models using induced pluripotent stem cells (IPSCs) and the future potential of gene therapy to directly replace or modify specific genes have kept inherited arrhythmic syndromes in the forefront of research and scientific discovery efforts. In this review, we discuss the basic principles and applications of genetic testing and potential future avenues for two of the best-understood conditions of this nature: long QT syndrome (LQTS) and catecholaminergic polymorphic ventricular tachycardia (VT) (CPVT).

## Use of genetic analysis in inherited arrhythmic syndromes

Genetic testing for both LQTS and CPVT is a class 1 indication.^1^ Although ordering genetic testing has become easier, choosing the right test and then interpreting the genetic results requires a high degree of specialist knowledge such that a multidisciplinary approach is advisable. The patient should be counseled in detail by an experienced genetic counselor or clinician regarding options, possible outcomes, and how the results may impact clinical management. The patient should understand that many genetic results will be of uncertain significance and should always be interpreted in conjunction with their clinical test results and not in isolation. The goal of genetic testing is twofold: (1) to identify specific variants in known disease-associated genes that further characterize the diagnosis in the proband and (2) to use this information to determine which family members are at increased risk of developing a disease and so require longitudinal cardiovascular evaluation as well as to identify relatives who do not require long-term follow-up. However, to define a variant as pathogenic or not, it needs to be systematically analyzed within the context of the familial phenotype to best define its causative impact.

## Basic genetic concepts

**Figure 1:**
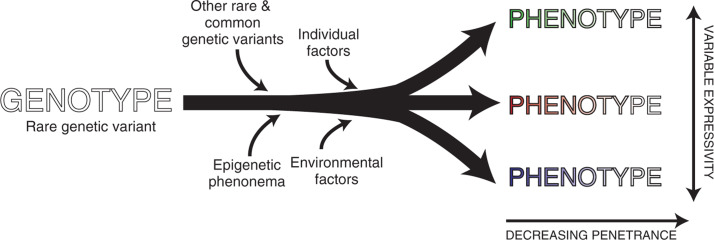
Relationship between genotype and phenotype. A rare genetic variant with a large effect (genotype) leads to a phenotype with varying degrees of penetrance (fading colors) and expressivity (different colors). This relationship is affected by many other factors and is fundamentally predicated on the accuracy of sequencing and interpretation of the genetic variant and identification of clinical manifestations.

In most cases, both LQTS and CPVT are inherited in an autosomal dominant pattern, but much rarer autosomal recessive variants have also been well-documented. Penetrance is typically less than 100%; that is, the number of individuals with a genetic predisposition to develop the condition is higher than the number of individuals who display the phenotype of that condition. Importantly, the identification of disease-causing variant(s) confers an increased risk of developing a clinical phenotype but does not equate to a clinical diagnosis.^2^ True nonpenetrance can make the inheritance pattern difficult to discern and should prompt careful consideration of whether this is truly the causal variant. Variable expressivity refers to individuals with the same underlying genetic predisposition to a disease who manifest different disease features and is best exemplified by the cardiac sodium channel gene *SCN5A*, where family members with the same mutation may have different phenotypes, such as Brugada syndrome, long QT syndrome, and conduction system disease. Penetrance and expressivity vary significantly within families due to impacts from a variety of other factors including other genetic sequence variations, environmental factors, and epigenetic phenomena^3^ **(Figure 1)**. See **Table 1** for more information on terms discussed here and elsewhere.

## Diagnostic genetic testing and interpretation

Successfully identifying the key person in the family in whom to initiate genetic testing and then selecting the correct test to perform optimizes the diagnostic yield and clinical utility of the result(s). Ideally, the patient with the most severe form and/or earliest onset of disease in the family should be the testing proband.^4^ The choice of the correct test can be complicated by overlapping phenotypes and genetic heterogeneity, where a similar phenotype can be produced by mutations in different genes. The provider should aim to identify a genetic panel that maximizes the yield while minimizing the likelihood of receiving an uncertain or ambiguous result.^5^

**Figure 2:**
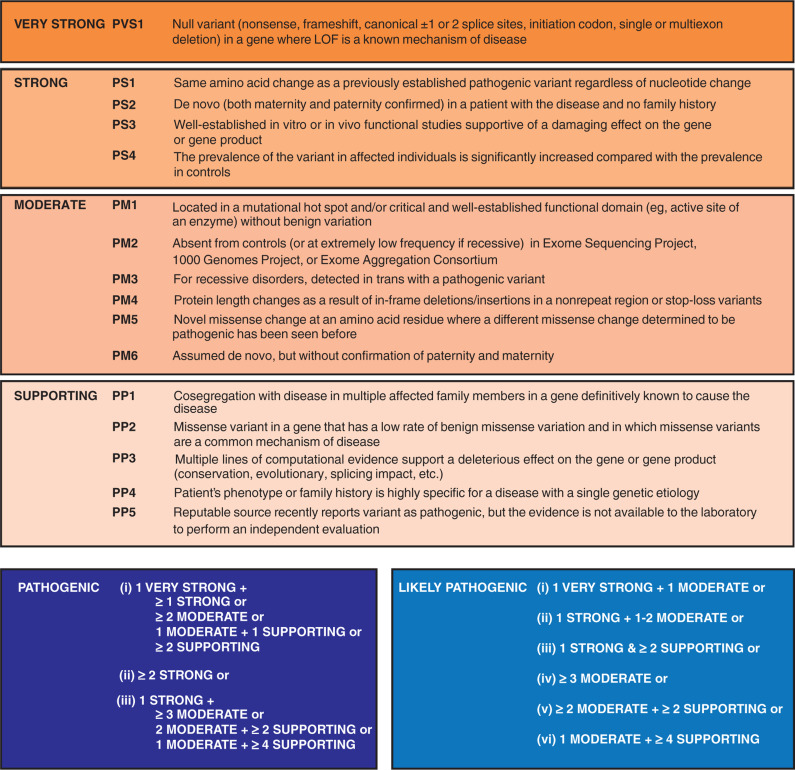
Criteria proposed by the ACMG for the interpretation of sequence variants. Depicted are specific variant characteristics, the categorization of which leads to the determination of pathogenic or likely pathogenic status. Variants that do not fulfill these criteria are considered to be of unknown significance or benign. For full details, see Richards et al.^6^

Perhaps the most compelling evidence to support variant pathogenicity is segregation with the phenotype across multiple family members, which can be described statistically using a logarithm of odds score. A logarithm of odds score > 3 (ie, there is a < 1:1,000 probability that the association is random) is typically considered as very strong evidence for variant causality in a given family. However, as sufficiently large pedigrees (with more than 10 family members characterized) are rarely available, other criteria are typically used. Depending on the level of evidence available, a genetic variant can be characterized on a continuum as benign, likely benign, uncertain significance, likely pathogenic, or pathogenic. The criteria to support each category were defined by the American College of Medical Genetics and Genomics (ACMG) in 2015 **(Figure 2)** in an attempt to standardize variant analysis and aid in interpretation.^6^ A pathogenic result supports a clinical diagnosis and may provide prognostic or therapeutic guidance^7^ as well as be impactful for cascade screening in family members. A benign variant is classified based upon prevalence in the general population or insufficient evidence to associate the gene or mutation type with disease. When there is insufficient evidence to categorize a variant as benign or pathogenic, it is termed a variant of uncertain significance (VUS). With rare exceptions, a VUS cannot be used to guide management in the proband or provide predictive information for asymptomatic family members.^8^ Classifications are dynamic and may change over time.

## Challenges in interpretation

Genetic testing should be used in individuals with either a clinical diagnosis or a strong clinical suspicion of a specific hereditary arrhythmic syndrome such as LQTS or CPVT. Testing an unaffected individual or a patient with a poorly defined phenotype will be less informative in general due to a low a priori risk. A low pretest probability correlates to a low positive predictive value for the test, even if the test is highly sensitive and specific.^8^ In other words, a priori risk is the likelihood that a patient has a condition before the test results are known; this is usually calculated based on pretest data including phenotype, sex, ethnicity, and family history. Commercial laboratories have different pipelines for analyzing and classifying variants, creating the potential for discordant interpretation of the same variant^9,10^; therefore, the onus is on the provider to analyze critically the given evidence for the variant themselves and correlate it with the phenotype in the proband and extended family. Ultimately, detailed phenotyping of the wider family is the most powerful tool to determine pathogenicity.^5^ There are multiple examples of variants originally deemed pathogenic failing to match with disease phenotype in family members, discrediting them as causative variants.^11^

A negative genetic test result does not exclude a diagnosis, particularly if the proband has a clinical diagnosis. It also does not rule out the possibility that the proband’s disease is hereditary; first-degree relatives should still be clinically screened appropriately based on the proband’s diagnosis. The likelihood of an actionable result is fundamentally predicated on the accuracy of the phenotyping and the specific test or panel ordered. For example, incomplete phenotyping could misguide the provider into ordering a targeted LQTS panel after exertional syncope, when the proband actually has CPVT and harbors a pathogenic variant in the cardiac ryanodine receptor (*RyR2*).^12^ Therefore, thorough phenotyping and detailed three-generation family history-taking are essential to informing the provider’s choice for the correct genetic test to order so as to avoid false negatives. Additionally, technology is rapidly advancing and novel disease genes are frequently added to panels. Retesting a previously negative individual if the sensitivity of the test used has increased since may be warranted.

## Familial predictive testing

If an affected proband is found to harbor a pathogenic variant, that result can be used to contribute to risk assessment in unaffected family members. Family members can be offered targeted testing specifically for the familial variant instead of repeating an entire panel, unless there is bilineal risk present in the pedigree or a family member has a phenotype that cannot be explained by the familial variant alone. Careful counseling regarding the risks, benefits, and limitations of genetic testing is essential to ensure that asymptomatic or presymptomatic family members are making informed choices and that expectations are properly managed, particularly given the potential for adverse psychological ramifications for the patient and the family members.^13^ Relative risk is a highly complex and abstract concept that many people struggle to comprehend,^14^ and genetic tests return probabilistic, not binary, results regarding the likelihood of developing a given disease^15^ without prognostic information such as age of onset or disease severity. In the case of pathogenic familial variants, a negative result returns the individual’s risk back to the population level.

Special consideration is necessary when the patient in question is a minor; the decision to pursue testing should be shared by the patient, parent or guardian, and provider to preserve patient autonomy and to ensure the best interests of the child. A child psychologist can facilitate the involvement of the child in this decision by presenting age-appropriate information and attending closely to issues of developmental characteristics that may impact children’s understanding or decision-making. Unfortunately, a lack of attention to these details can result in resentment and noncompliance in the child and anxiety and guilt in the parent.

## Long QT syndrome

### Background

A disorder of ventricular myocardial repolarization, LQTS is characterized by QT prolongation and morphological T-wave abnormalities that predispose to ventricular arrhythmias and clinically manifest as syncope, cardiac arrest, and sudden cardiac death. LQTS is caused by genetically encoded abnormalities in sodium, potassium, and calcium cardiac ion channels. The autosomal recessive form associated with sensorineural hearing loss was first reported by Jervell and Lange-Nielsen in 1957^16^ and the dominant form was subsequently described by Romano in 1963 and Ward in 1964,^17,18^ respectively. A clinical and genetic analysis of 45,000 neonates reported in 2009 suggested the prevalence of LQTS to be in the range of 1:2,000 to 1:2,500.^19^ The diagnosis of LQTS is based on a variety of personal, electrocardiographic, familial, and genetic criteria.^20^

### Inheritance and penetrance

LQTS is typically inherited in an autosomal dominant manner with highly varied penetrance; however, compound heterozygous (two variants within one gene), digenic (variants within two different genes), and homozygous (the same variant in both alleles of a single gene) cases have all been recognized. Cases with more than one variant in *KCNQ1* may be associated with sensorineural deafness.^21^ Before genetic testing, detailed phenotypic evaluation of the proband and wider family can help to determine the LQTS type and inheritance pattern(s) and guide the interpretation of any identified variants. Although LQTS may occur de novo, a lack of any apparent family history is much more likely to be related to clinically quiescent disease. Disease penetrance may be as low as 25%^22^; hence, a normal resting electrocardiogram (ECG) does not exclude the condition in first-degree family members. Abnormal T-wave morphology and sinus bradycardia are well-recognized^23^ and, in both the LQTS1 and LQTS2 types, the phenotype may be unmasked with simple maneuvers such as standing and exercise.^24^

### Genetic basis

The underlying genetic defects of long QT syndrome were first described in the 1990s, identifying three genes (*KCNQ1*, *KCNH2*, and *SCN5A*) that encoded ion channel proteins (KvLQT1, hERG, and NaV 1.5) responsible for transmembrane ion currents critical to cardiac depolarization and repolarization. Clinically, they are referred to as LQTS1, LQTS2, and LQTS3. Structural and functional protein abnormalities lead to either excessive sodium (*I*_Na_) influx and persistent depolarization or delayed potassium (*I*_Ks_ and *I*_Kr_) efflux and prolonged repolarization, the net effect of which is prolongation of the myocardial action potential duration and the QT interval on the surface ECG. *KCNQ1*, *KCNH2*, and *SCN5A* remain as the most prevalent genes and are the focus of this current review. Variants in other cardiomyocyte ion channels or assembly proteins have been implicated in monogenic LQTS but, recently, the ability of variants in the beta subunit KCNE2 to cause disease in the absence of environmental or other genetic factors has been questioned.^25^ The contribution of genes involved in cellular calcium homeostasis is increasingly recognized: for example, *CACNA1C* underlies the multisystem Timothy syndrome, while the three calmodulin genes (*CALM1*, *CALM2*, *CALM3*) and triadin (*TRDN*) may cause highly malignant forms of LQTS.

### Interpretation of genetic test results

**Figure 3:**
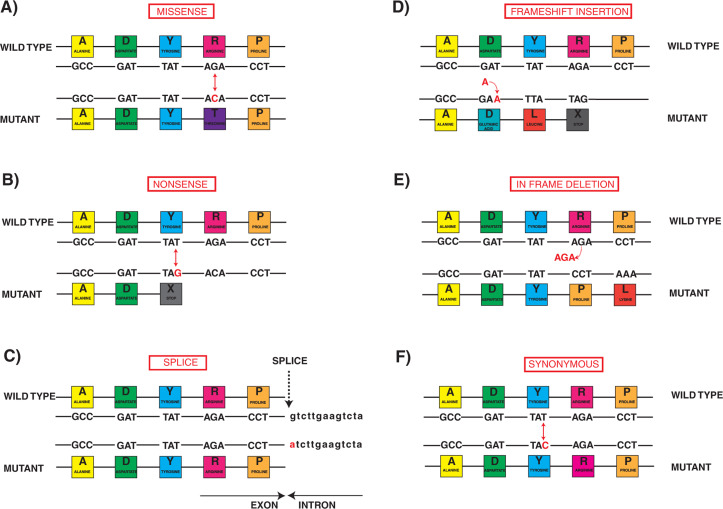
Different consequences of genetic sequence variation. Displayed in each panel is the wild-type sequence with specific nucleotide variation and the subsequent amino acid consequence. **A:** Missense: a single nucleotide point variant (G to C) changes the amino acid from arginine to threonine. There is no change in the downstream reading frame or amino acids. **B:** Nonsense: another single-point variant (T to G) changes tyrosine to a premature stop codon and protein truncation. **C:** Splice variant: the nucleotide coding sequence and amino acid sequence within the exon are normal, but the first nucleotide in the intron has changed (G to A). Spliceosomes depend on the nucleotide sequence at exon–intron boundaries and, if variation in the nucleotide sequence occurs in the canonical splice sites at the exon–intron boundaries, such variation can lead to abnormal splicing and either exonic skipping or intronic inclusion. **D:** Frameshift insertion: a single nucleotide (A) is inserted (dashed arrow) and hence alters the downstream reading frame and typically leads to a stop codon and premature truncation, in this case after only one further amino acid residue. **E:** Inframe deletion: three nucleotides (AGA) and a single amino acid (arginine) are deleted (dashed arrow), but the prior and subsequent amino acid sequence portions are unaffected. Inframe nucleotide insertions or deletions (indels) occur in multiples of three beginning at the first nucleotide of an amino acid residue, whereas frameshift indels do not and therefore alter the subsequent reading frame. **F:** Synonymous variant: a single-point mutation (T to C) occurs, but the two nucleotide triplets (TAT and TAC) both encode the same amino acid, tyrosine. Synonymous variants may be benign, though, if located in the terminal nucleotides of the exon, such can lead to abnormal splicing. Synonymous variants have been identified in LQTS1 and LQTS2.

Genetic testing reports include the gene; nucleotide substitution; amino acid effect; zygosity; consequence of the sequence code variation (ie, missense, insertion/deletion, frameshift, splice site, or truncating) **(Figure 3)**; and a determination of pathogenicity based on variant characteristics coupled with prior reports including segregation, functional studies, variant prevalence in control databases, and in silico analysis. In silico analytic tools such as PolyPhen-2 (Polymorphism Phenotyping version 2; Sunyaev Laboratory, Harvard Medical School, Boston, MA, USA) or MutationTaster (Jana Marie Schwarz and Dominik Seelow, Charité – Universitätsmedizin Berlin, Berlin, Germany) are computer-based algorithms used to estimate pathogenicity based on the predicted impact of the specific nucleotide and amino acids changes on protein structure and function. Many important factors should be considered when determining the potential causal relationship of any specific variant with LQTS, such as whether the clinical phenotype can be explained by the identified genotype. For example, an *SCN5A* variant in a 15-year-old with exertional syncope, a corrected QT (QTc) interval of 480 ms, and significant QTc prolongation in early recovery on an exercise test is unlikely to be causal, whereas a variant in *KCNQ1* is much more consistent with the clinical picture. This may have increasing importance if variants in more than one gene are identified.^26^ Although the loss-of-function variants (eg, truncation, splice, frameshift insertion, deletion) are more likely to be categorized as pathogenic by the 2015 ACMG classification as compared with missense mutations, *KCNQ1* may be highly tolerant to the loss of one functional allele, a factor calculated by the number of observed versus expected losses of function variants in population databases.^27^ Conversely, both *KCNH2* and *SCN5A* are highly intolerant to loss of function.^27^

The topological location of the variant may add further information, with specific areas showing high degrees of conservation across species and an enrichment of pathogenic variants. Cellular electrophysiology studies using transfected cell lines such as *Xenopus* oocytes or human embryonic kidney cells can help to determine the consequences of genetic variation on ion channel function. However, variant location and in vitro effects are not always concordant with the associated clinical phenotype.

**Figure 4:**
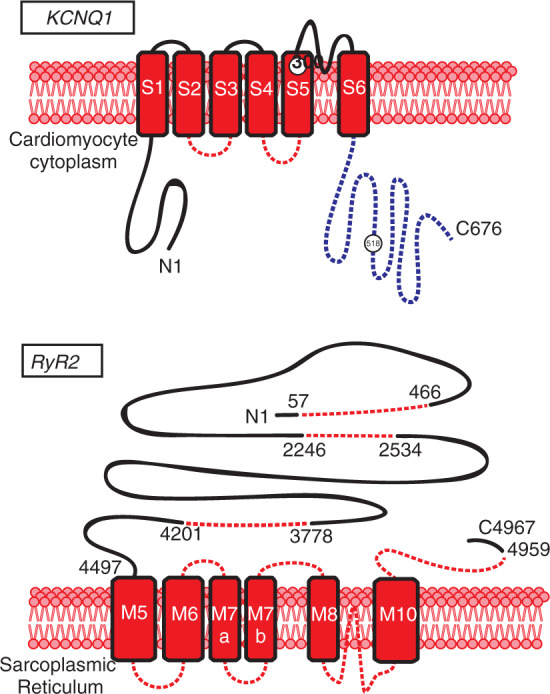
Topology of the genes *KCNQ1* and *RyR2*. The upper panel depicts *KCNQ*1 associated with LQTS1. The cytoplasmic loops are depicted as the red dashed line and the C-terminal is depicted as the blue dashed line. The positions of amino acid residues 300 in the pore helix and 518 in the C-terminal are shown. The lower panel depicts *RyR2* associated with CPVT. The four canonical domains where the majority of disease-associated variants are located are depicted as a red dashed line, along with the first and last amino acid residues depicted numerically. See the text for further details.

To illustrate, consider a variant in *KCNQ1*, A300T **(Figure 4)**. If identified in a patient, a report may conclude that this variant (1) is located in the pore helix (AA 298-312) region, which shows a high degree of conservation and enrichment for pathogenic variants due to the fundamental role of the regions in channel function^28^; (2) reduces *I*_ks_ to 15% of the wild type according to functional assessment^29^; and (3) has been previously associated with long QT syndrome.^30^ Upon review of such information, it would appear that the variant would highly likely lead to clinically severe and manifested LQTS. However, a more detailed review of the prior case would reveal that LQTS associated with A300T is apparent only in homozygous, not heterozygous, carriers,^30^ and the variant has been identified in healthy controls (with a minor allelic frequency of 0.003%). Although the variant was predicted to be pathogenic by in silico analysis and absent from the Exome Aggregation Consortium database, the diagnosis was reversed based on detailed clinical assessment of the family and more detailed molecular modeling. Similarly, the *KCNQ1* splice variant c.477+1G>A has been associated with a phenotype in homozygous but not heterozygous individuals. Although detailed functional analysis suggested an effect should be seen in heterozygote carriers, natural degradation in vivo of the abnormal messenger ribonucleic acid (RNA) (ie, nonsense-mediated messenger RNA decay) prevents production of the mutant protein and limits expression of LQTS.^31^

De novo *SCN5A* variants with severe functional consequences have been identified in neonates with severe QT prolongation and recurrent ventricular arrhythmias.^32^ Such variants, absent from the majority of population databases, promote early symptomatic presentation due to the severe proarrhythmic disruption of cellular depolarization. Several other *SCN5A* variants identified as part of the detailed sequencing of arrhythmia susceptibility genes in a sudden infant death cohort^33^ demonstrated a marked increase in the late sodium current (*I*_Na_) when expressed in tsA201 cells consistent with a LQTS phenotype; in two, S216L and T1304M, the persistent *I*_Na_ was > 1%,^34^ but both of these variants have been identified in racially concordant population databases at mean allelic frequencies of 0.03% and 0.15%, respectively.^27^ Considering that the population prevalence of LQTS is 1:2,000 (0.05%), this suggests that both of these variants are too common to be considered causal for LQTS in every case, but their potential arrhythmic role under the influence of other genetic or environmental stressors is unknown. Conversely, an *SCN5A* variant associated with atrial standstill, conduction abnormalities, and ventricular dysfunction has been found not to disrupt channel function in Chinese hamster ovary cells but did recapitulate the phenotype in a mouse model.^35^

### Genotype-guided treatment strategies

An important consideration is to what degree can or does the genotype impact clinical decision-making. Ultimately, clinical management should be governed by the phenotype, but knowledge of the underlying genetic variant can help to guide clinical management in specific circumstances. β-blockers, specifically propranolol and nadolol, remain the backbone of LQTS treatment; however, in the absence of any randomized controlled trials, it is unclear as to whether every patient identified by cascade screening with lowly penetrated forms of the disease requires treatment. Although β-blockers can be considered an “insurance policy,” they are not without side effects and, in the absence of any phenotypic expression on detailed clinical testing, their overall long-term benefit is unclear. Many patients take β-blockers intermittently or not at all,^36^ with the former raising concern for receptor upregulation and elevated risk.^37^

The *KCNQ1* R518X Swedish founder variant has been extensively studied and is associated with a relatively mild phenotype in heterozygous carriers.^38,39^ In light of this, one interesting question to consider is whether or not an asymptomatic adult male with a normal resting QT interval on repeated measurements should take a β-blocker or whether avoidance of QT-prolonging medications is sufficient in this individual. Conversely, mutations in the two cytoplasmic loops (C-loops) S2-S3 (AA171-195) and S4-S5 (AA242-262) appear to confer a significantly higher risk of life-threatening cardiac events in comparison with variants in other topographic locations, an effect significantly negated by β-blockade.^40^ Owing to initial concerns about the proarrhythmic effects of bradycardia and higher rate of breakthrough cardiac events,^41^ patients with *SCN5A*-mediated LQTS3 are typically treated more aggressively with implantable cardioverter-defibrillators (ICDs). This notion was refuted by a more recent study of 391 LQTS3 patients, which found β-blockers significantly reduced the risk for cardiac events, especially in females.^42^ Mexiletine was recently shown to have beneficial effects in reducing both QT duration and frequency of cardiac events in LQTS3 patients, either in conjunction with β-blockade or in isolation.^43^

## Catecholaminergic polymorphic ventricular tachycardia

### Background

CPVT typically presents in childhood with symptoms of palpitations, syncope, seizures, and cardiac arrest during exertion or emotional stimuli.^44^ Although symptoms are typically precipitated by exercise or emotion, both syncope and cardiac arrest may occur during normal activities and rest.^45^ First recognized in the 1960s, it was not until 1978 that Phillippe Coumel and colleagues in Paris recognized the adrenergic basis and specific ECG pattern and subsequently in 1995 reported their experience of 21 patients, proposing the descriptive term “catecholaminergic polymorphic ventricular tachycardia.”^46^ The hallmark ECG marker is bidirectional VT caused by triggered afterdepolarizations thought to arise in an alternating fashion from the His-Purkinje system in the right and left ventricles, giving rise to the classical appearance.^47^ Although the resting ECG is normal, sinus bradycardia is common^48^—as is atrial fibrillation,^49^ which may predate ventricular arrhythmias. Bidirectional VT may also be seen in Andersen-Tawil syndrome (ATS). A multisystem ion channel disorder previously referred to as LQT7, ATS is associated with a prolonged QU interval and prominent U waves on the surface ECG, a high burden of ventricular ectopy often at rest, syndactyly, characteristic dysmorphic facies, and periodic paralysis.

### Inheritance and penetrance

CPVT most commonly displays an autosomal dominant inheritance pattern with a high degree of penetrance, although probands typically show a more severe disease phenotype than do family members identified through cascade screening. The detailed evaluation of family members including one family with 61 individuals (*RyR2* R420W) revealed a more subtle or absent phenotype in many. Interestingly, 50% of family members with a normal initial clinical evaluation may develop disease features, stressing the need for ongoing evaluation.^50^ Absence of clinical features in family members may also point to de novo disease occurrence, which may be more common than familial disease,^51^ the much rarer autosomal recessive CPVT,^52^ or germline mosaicism.^53^

### Genetic basis

CPVT is caused by genetically encoded abnormalities in cardiomyocyte proteins fundamental to cardiomyocyte calcium homeostasis, most commonly in *RyR2* and associated proteins calsequestrin (*CASQ2*), calmodulin (*CALM*), and *TRDN*. Approximately 60% to 70% of patients with a definitive clinical diagnosis of CPVT will have identifiable variants in *RyR2*, with most located in one of the following four highly conserved, functionally important domains of the gene: domain I AA 57-466, domain II 2246-2534, domain III 3778-4201, and domain IV 4497-4959 **(Figure 4)**. Most *RyR2* variants associated with CPVT are missense or small insertions/deletions as opposed to truncating variants, supporting a dominant negative mechanism wherein the mutant protein prevents normal function of the wild type.^52^ A large inframe deletion incorporating exon 3 and associated intronic sequences has been linked with a broader phenotype of left ventricular noncompaction, conduction disease, atrial arrhythmias, exertional ventricular arrhythmias, and sudden death. The deletion (c.161-236_c.272+781del1126; p.Asn57_Gly91del) segregated with the phenotype [logarithm (base 10) of odds score: 4.5] in one family and is framed by two *Alu*-repeat sequences frequently associated with genomic rearrangement.^54^ Although the clinical phenotype and associated deletion have been identified in other unrelated cases, murine models of exon 3 deletion do not replicate the phenotype^55^ and the exact mechanism of how this sequence variation leads to the more diverse and complex phenotype remains unclear.

*CASQ2*-mediated disease is almost exclusively related to homozygous or compound heterozygous variants involving both truncating and missense mutations.^56^ Heterozygote carriers are typically unaffected, but recently, a heterozygote variant was shown to segregate [logarithm (base 10) of odds score: 3.01] with an overt CPVT phenotype in a family with autosomal dominant inheritance.^57^ The calmodulin genes (ie, *CALM1, CALM2*, and *CALM3*), *TRDN*, and trans-2,3-enoyl-CoA reductase-like protein (TECRL) may all lead to CPVT or LQTS, often with phenotypic overlap.^58–62^ *KCNJ2*, which encodes Kir2.1, the inward rectifier potassium channel, is associated with ATS but may also produce an isolated CPVT phenotype, clinically distinguishable from other variants by the presence of ventricular arrhythmias and complex ectopy at rest.^63^

### Interpretation of genetic test results

The interpretation of variants identified in CPVT is challenging. *RyR2* has almost 5,000 amino acid residues, which, in combination with the rarity of CPVT, means that many patients are found to carry novel missense variants, appropriately classified as VUSs. A de novo variant in a child with CPVT is more likely to be disease-causing with confirmed maternity and paternity and normal clinical parental evaluation. Therefore, significant care needs to be applied in interpreting *RyR2* variants as disease-causing; an analysis of the Exome Sequencing Project database of 6,503 control subjects identified 41 missense variants previously associated with CPVT, which, if truly causal, would lead to a disease prevalence of 1:150, not the estimated value of 1:10,000.^64^ Notably, in this study, no variants were identified in the four canonical domains critical to protein function and, in comprehensively phenotyped CPVT cohorts, most variants, but not all, locate to one of these four canonical domains.^44,51^ However, variants within these domains have also been identified within larger control databases and patients undergoing whole exome sequencing for noncardiac indications,^65^ suggesting that, although these canonical domains may be highly enriched with true disease-causing variants, a diagnosis of CPVT cannot be made based on the topographical location of a genetic variant alone and careful evaluation in the context of the phenotype and pretest probability is required.

### Genotype-guided treatment strategies

Given the association of symptoms with catecholaminergic stress, β-blockers have been the mainstay of treatment, although, more recently, flecainide^66^ and left cardiac sympathetic denervation^67^ have shown beneficial effects. Furthermore, although ICDs are important components of care, interest in their use has significantly declined due to the limited effects of defibrillation in the management of arrhythmias caused by triggered activity; the well-recognized complications, including death, of ICD use in a young population; and the specific proarrhythmic complications seen in patients with CPVT.^66^ At present, the associated genetic variant identified in a patient with CPVT does not directly impact clinical management.

## New avenues

Technological advances in the use of cardiomyocytes derived from IPSCs (IPSC-CMs) have allowed for the creation of patient-specific models for both LQTS and CPVT, providing further insights into the therapeutic effects of different pharmacological agents and disease biology. Although such models do not completely recapitulate the in vivo biology of mature human cardiomyocytes, they have many advantages over animal models and transfected cell lines. IPSC-CMs incorporating both rare variants with large effects and other single-nucleotide polymorphisms correlate well with the molecular and arrhythmic phenotypes seen in both LQTS and CPVT, which proposes the interesting possibility of human-derived models for variant testing. Commercially available IPSC-CMs transfected with a clinically identified *KCNJ2* mutation have been shown to recapitulate the phenotype identified in the patient, supporting causality for the variant in question.^67^ At present, such analysis is time-consuming and costly but, as gene editing technology improves, the potential for using IPSC-CMs to determine the effects of specific variants will become increasingly recognized.

In the last three to four years, the possibility of gene therapy for inherited cardiovascular disorders has moved closer to reality, specifically by overexpression of the target protein in autosomal recessive CPVT and by silencing of the mutant allele in the dominant form. In murine models of homozygous *CASQ2* R33Q^−/−^ CPVT, in vivo delivery of an adeno-associated virus complementary DNA *CASQ2* (AAV-*CASQ2*) construct was performed in three-day-old and three-month-old knock-in animals. A single injection was able to prevent the development of CPVT in the younger mice and revert the phenotype in the older, symptomatic mice who had already demonstrated either polymorphic or bidirectional VT in response to epinephrine. In both groups, there was a restoration of the physiological levels of calsequestrin and associated proteins junctin and triadin, with normal protein–protein interactions, as well as the prevention or restoration of ultrastructural cellular changes. Although only 40% of myocardial cells were infected with the AAV9-*CASQ2* construct in both the neonatal and the older mice, interestingly, this was sufficient to confer a significant antiarrhythmic benefit, an effect attributed to the presence of sufficient rescued cells to prevent widespread transmission of delayed afterdepolarizations.^68^ Subsequent studies have been able to replicate the beneficial effects of AAV-*CASQ2* delivery in IPSC-CMs obtained from a patient with CPVT secondary to a homozygous *CASQ2*-truncating mutation.^69^ In animal models of *RyR2*-mediated CPVT, a different approach is necessary to suppress the dominant negative effects of the mutant protein. Allele-specific silencing using adeno-associated virus small interfering RNA duplexes has been shown to suppress the mutant *RyR2* messenger RNA (*RyR2*-R4496C) with no significant effect on wild-type levels, correlating with a reduction in delayed afterdepolarizations, ventricular arrhythmias, and ultrastructural abnormalities in treated animals.^70^

## Conclusions

Since the seminal discovery 30 years ago of the genetic abnormalities that underlie inherited cardiac arrhythmias, our understanding of the complexities of monogenic disorders such as LQTS and CPVT has advanced parallel to technological changes that have supported deeper investigations into the associated genetic and molecular pathways. It is increasingly clear that the relationship between rare genetic variants and associated phenotypes is far from linear, influenced by multiple factors, which thus far remain beyond the scope of day-to-day clinical practice. Consequently, care should always be taken in the interpretation of identified genetic variants, with detailed phenotypic evaluation of the wider family used to validate genetic findings and dictate clinical management. As technology continues to advance at a rapid pace, the opportunities for creating patient-specific models to better define different genetic influences on cardiomyocyte biology will become greater and the possibility of ultimately correcting the underlying genetic sequence variation of inherited cardiac disorders will grow closer.

## Figures and Tables

**Table 1:** Glossary of Terms

A priori risk	The pretest probability that an individual will harbor a pathogenic variant as determined by numerous factors including the individual’s phenotype, family history, previous genetic testing, and clinical sensitivity to the test.
Bilineal risk	Describes the scenario wherein an individual has potentially inherited a genetic predisposition to disease from both parents.
Canonical splice site	The specific base-pair sequence where splicing most frequently occurs, namely the first (GT) and last (AG) intronic nucleotides. These are referred to as the donor and acceptor sites, respectively.
Codon	A triplet of three nucleotide base pairs of DNA that encode a specific amino acid.
Compound heterozygote	A genotype in which both alleles of a specific gene are mutated at different loci.
De novo	A term used to describe a spontaneous mutation versus one that was inherited from a parent. To establish that a variant is truly de novo, studies confirming maternity and paternity of the patient should be performed.
Digenic inheritance	A term that refers to a single disease caused by mutations in two unique genes in the same individual.
Dominant negative	A phenomenon in which the mutant allele disrupts the function of the wild-type allele in the same cell.
Epigenetic	A term that refers to factors that can influence gene function without altering the genotype. Examples of this include methylation, histone modification, and transcription factor alteration.
Expression	The degree to which a genetic mutation manifests as a clinical expression of a disease. For example, a mutation may cause a range of mild to severe disease symptoms in different individuals with the same mutation.
Genotype	The genetic constitution of an individual.
Haploinsufficiency	A mechanism of disease in which one functioning allele is not enough to prevent disease expression due to a loss of function in the other allele.
Heterozygote	An individual with a genotype of two different alleles at a specific locus.
Homozygote	An individual with a genotype of identical alleles at a specific locus.
In silico analysis	A computer-based modeling initiative that attempts to predict the impact of a nucleotide or amino acid change on the resulting protein structure and/or function to determine potential disease causality.
Penetrance	The fraction of individuals with a pathogenic mutation predisposing to disease who have physical evidence of the disease.
Phenotype	The physical evidence of disease expressed by an individual as a result of the underlying genotype and environmental factors, among others.
Proband	The first individual in a family to present with an expression of the disease.
